# Archived Historical Aquatic Macroinvertebrate Specimens Suggest Connections Between Microplastic Abundance Patterns, Trophic Traits, and Land Use

**DOI:** 10.3390/insects17040386

**Published:** 2026-04-02

**Authors:** Rachel E. McNeish, Marisa D. Macchia, Nicole M. Lee, Austin T. Harrison, Alexandra J. Brown, John K. Jackson, John R. Wallace

**Affiliations:** 1 Department of Biology, California State University, Bakersfield, Bakersfield, CA 93311, USA; 2 Department of Biology, Millersville University, Millersville, PA 17551, USA; 3 Stroud Water Research Center, Avondale, PA 19311, USA; jkjackson@stroudcenter.org

**Keywords:** plastic pollution, watershed, streams, insects, life stage

## Abstract

Plastic pollution is ubiquitous in the environment, with microplastics (particles < 5 mm) a worldwide emergent contaminant of concern; however, no studies have explored historical patterns in freshwater macroinvertebrates in connection to species traits and changes in land use. We measured microplastics in macroinvertebrates collected from 1998–2019 at six forest, urban, and agriculture dominated streams in the Schuylkill River watershed (PA, USA). We selected representative macroinvertebrates that underwent peroxide oxidation digestion and were examined for microplastics under a dissecting microscope with Rose Bengal dye to confirm microplastic identification. Microplastics were present in 134 individuals across 24 taxa and all functional feeding groups (FFGs). Abundance of microplastics in macroinvertebrates decreased through time with an increase of agriculture land use, whereas microplastics increased with an increase of urban land use. Collector-gatherer FFG had the greatest microplastic abundance, which may be linked to Elmidae and Psephenidae (Coleoptera), which tended to have the highest abundances of microplastics compared to all other taxa in some years. This relationship may be explained by the feeding mode of these families of macroinvertebrates and that gathering particles in the water column and scraping particles from biofilms may be similarly effective in the acquisition of microplastics. Overall, understanding microplastic temporal patterns and their connections to species traits and variations in land and water use is critical to developing a predictive framework for explaining microplastic abundance in the environment and interactions with aquatic biota.

## Simple Summary

Plastics are a global contaminant of concern due to their large accumulation and pervasiveness in the environment worldwide. The global abundance and distribution of plastic pollution have been under scientific investigation since the 1970s; however, the ecological importance of plastics in ecosystems has been understudied. Microplastics (particles < 5 mm) have garnered attention due to their integration into terrestrial and aquatic food webs that also includes contamination of human environments, food, and beverages. In this study, we measured microplastic abundance in aquatic macroinvertebrates from archived insect collections originating from six streams. We predicted that microplastic abundance in aquatic macroinvertebrates would be greater in urban and agriculture dominated watersheds compared to forested watersheds, in macroinvertebrate predators compared to other consumers, and that microplastic abundance would increase in macroinvertebrates through time. Findings from this study suggested that microplastic abundance varied among years and percent land use (i.e., percent urban, agricultural and forest) in the landscape, partially supporting our predictions. Results also demonstrated that microplastics are prevalent among various pollution tolerant and intolerant taxa. Archived historical specimens in this study highlighted the importance of considering feeding strategies and connections to changes across watershed landscapes when studying microplastic pollution in the environment and interactions with aquatic biota.

## 1. Introduction

Plastics are a global contaminant of concern due to their large accumulation and pervasiveness in the environment worldwide [1]. Total global plastic production reached 6300 million Mt (metric tons) by 2015 [2], with annual plastic production now more than 300 million Mt of plastics [3,4]. Plastic pollution has been documented in terrestrial [5,6], marine [7,8], freshwater [9,10], aquifer [11], atmosphere [12,13], and arctic [14,15] systems. Sources of environmental pollution with plastics to the environment include fragmentation of existing plastic litter [16,17], industrial production [18], and treated wastewater effluents [19,20]. The global abundance and distribution of plastic pollution have been under scientific investigation since the 1970s [21]; however, the ecological importance and cycling of plastics in ecosystems has been understudied until recently [22,23].

Microplastics (particles < 5 mm) [24] have garnered much attention due to their integration into terrestrial and aquatic food webs that include birds, fishes, aquatic mammals, and invertebrates [25,26,27,28,29], and contamination of human environments, food, and beverages [30,31,32,33,34]. Microplastics can serve as vectors for pathogenic microbes and heavy metals [35,36]. For example, *Aeromonas salmonicida* [37], a fish pathogenic bacterium known to cause furunculosis (tail rot), was identified on microplastics from the North Adriatic Sea [35], and Li et al. [36] demonstrated that microplastics from wastewater sewage sludge were greater in cadmium, copper, and lead heavy metals compared to the rest of the sewage sludge matrix, suggesting that microplastics may play an important role in the transport and fate of pathogens and heavy metals in the environment. Microplastics are also known to cause irritation to gut digestive tissues, reduce animal nutrient assimilation, and can contribute to the transfer of adsorbed persistent organic pollutants (e.g., DDT) to animal tissues and fluids [27,38,39,40,41]. However, some studies have demonstrated that microplastics may have minimal impacts on the health of some organisms [42]. In addition, trophic transfer of microplastics has been documented between algae–herbivore (i.e., seaweed to common periwinkle whelk) and predator–prey interactions [43,44]. The previously mentioned studies collectively demonstrate the potential for microplastics to have deleterious effects on organisms across multiple trophic levels, highlighting the need for more research to understand how environmental factors and species traits may explain microplastic abundance in organisms.

Insect and macroinvertebrate–microplastic interactions have garnered increased attention in recent years due to the ecological importance of invertebrates. For example, female fungus gnats (*Gradysia difformis* [Frey]; Diptera) were found to be less likely to oviposit on plant–soil systems exposed to microplastics, suggesting microplastics may indirectly alter the reproductive behavior of female insects [45]. Short-term exposure of polyurethane microplastics to Chironomidae larvae (*Chironomus riparis* [Meigen 1804]) resulted in no detected effects on insect growth [46]; however, other studies reported both increased and decreased effects of growth when exposed to microplastic polymer mixtures on chironomids *C. riparis* and *C. tepperi* (Skuse 1889), respectively [46,47]. Survivorship of common bluetail damselfly larva (*Ischnura elegans* [Vander Linden 1820]) was not impacted by polystyrene microplastics, but this exposure did result in intestinal inflammation of the larva, which might have contributed to the significant shift in the microbial gut flora community compared to larva not exposed to these microplastics [48]. A study conducted by Stankovic et al. [49] found that exposure to a high concentration of a polydisperse microplastic mixture was found not to impact pond macroinvertebrates functional feeding group biomass or community diversity, which was interesting, since Scherer et al. [50] reported that microplastic uptake by aquatic macroinvertebrates was linked to feeding strategy. These studies, among several others, collectively demonstrate that macroinvertebrates are exposed to, and are able to ingest, microplastics in their environments; however, the implications of microplastic pollution are varied and unclear, indicating the need for continued research on microplastic–insect and –macroinvertebrate interactions.

Anthropogenic activities and changes in land and water use play a critical role in the terrestrial–aquatic linkages associated with the abundance, transport, and fate of natural and anthropogenic materials to riverine systems and interactions with components of these systems [10,51,52,53,54,55,56,57]. For example, human-dominated landscapes often result in pharmaceuticals and personal care products entering freshwaters due to failing infrastructure (e.g., sewage leakage), wastewater treatment plant (WWTP) effluents, anthropogenic activities, and surface water runoff [58,59,60] that can impact aquatic biota and ecosystem function [61]. Agriculture croplands are known to be non-point sources of plastic pollution in the environment [62]. For example, Mahon et al. [62] found biosolids from human sewage sludge had an average microplastic abundance from 4196 to 15,385 particles kg^−1^ dry mass across seven WWTP sites, suggesting that the application of these biosolids as fertilizers to croplands contributes to the movement of microplastics from WWTPs to croplands to nearby aquatic habitats during precipitation (runoff) events. Riparian vegetation plays a key role in promoting freshwater water quality and supports aquatic biota by acting as a nutrient sink and mediating nutrient pollution from the terrestrial landscape to freshwaters [63,64,65,66]. Understanding how changes in land use and landcover (LULC) impact terrestrial–aquatic connections has proven to be pivotal for managing freshwater habitats; therefore, it is important to understand how LULC may be connected to plastic pollution in rivers and streams.

In this study, we measured microplastic abundance in aquatic macroinvertebrates from archived insect collections originating from six headwater streams that spanned a LULC gradient in the Schuylkill River watershed of southeastern Pennsylvania, USA, across three decades. Our objectives included (1) the identification and quantification of microplastics among different insect taxa and life stages; (2) a comparison of microplastic abundance among different functional feeding groups (e.g., shredders, filter feeders, grazers, predators); (3) a determination if microplastic abundance in examined individuals differed among land use types (e.g., forested, agriculture, urban); (4) a determination if microplastic abundance in different individuals differed over different time periods (1999, 2010, 2019) and; (5) a comparison of whether microplastic abundance in laboratory controls differed from abundance of microplastics found in preserved macroinvertebrates. We predicted (*P*_1_) that both aquatic insect larvae and pupae of the same insect taxa will have microplastics, with microplastic abundance reduced in pupae compared to larvae. We predicted (*P*_2_) that microplastic abundance would be greater in higher functional feeding group (FFG) trophic positions (i.e., predators) compared to lower FFG trophic positions (i.e., consumers of algae [grazers] and detritus [detritivores]). We predicted that microplastic abundance in aquatic macroinvertebrates would be greater (*P*_3_) in urban and agriculture-dominated watersheds compared to forested watersheds, and that (*P*_4_) microplastics abundance would increase in macroinvertebrates through time. Finally, (*P*_5_) we expected that control samples from the laboratory would contain less microplastics and be distinct in morphology and color compared to preserved macroinvertebrate samples. Our study contributes knowledge to the growing body of macroinvertebrate–microplastic literature by demonstrating a novel use of archived macroinvertebrate species while exploring the intersection of macroinvertebrate foraging and feeding traits, microplastic temporal patterns, and LULC linked with microplastic abundance in aquatic macroinvertebrate communities, which has not been previously explored.

## 2. Methods and Materials

**Figure 1:**
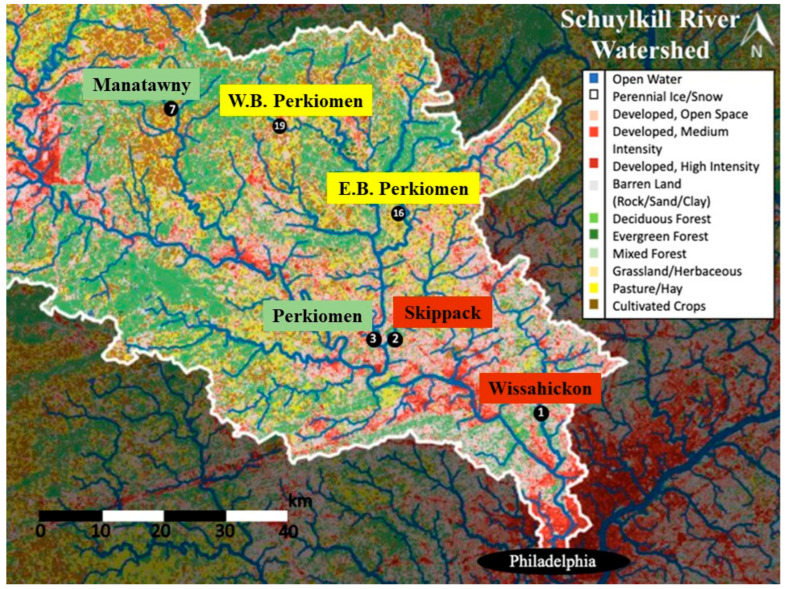
Map of the six study streams located in the Schuylkill River Watershed in southeastern Pennsylvania, USA. Urban streams included Skippack Creek and Wissahickon Creek; Agricultural streams included West Branch Perkiomen and East Branch Perkiomen; Forested streams included Perkiomen Creek and Manatawny Creek.

In 1996, the Stroud Water Research Center began an on-going study designed to examine water quality changes using aquatic macroinvertebrates as indicator taxa from 19 sites along the major waterways of the Schuylkill River Watershed, including varying physiographies and land uses (Figure 1; [67]). The current study has taken a subsample of six of these sites to provide a historical perspective of the microplastic abundance within this watershed.

### 2.1. Field Sites

Six headwater streams as part of an ongoing, long-term monitory study within the Schuylkill River watershed in southeastern Pennsylvania, USA, which included a mixture of land and water use spanning a gradient of percent agriculture (East and West Branch Perkiomen Creeks), urban development (Skippack and Wissahickon Creeks), and forest (Perkiomen and Manatawny Creeks; Table 1; Figure 1) were selected. The dominant LULC was determined by calculating the relative abundance of urban, forest, and agriculture in each stream’s sub-watershed using the 2011 National Land Cover Dataset in Model My Watershed Program [68,69]. All fieldwork was conducted with permission from state and local officials and private citizens.

### 2.2. Macroinvertebrate Sampling

We subsampled study organisms from aquatic macroinvertebrate (i.e., aquatic insects and other aquatic invertebrates) specimens collected in 1998, 2010, and 2019 archived at the Stroud Water Research Center in Avondale, PA, USA. During those years, original collections were randomly sampled from a single riffle habitat at each stream site using a modified Hess sampler (0.088 m^2^ sample area; 500 µm mesh net). Benthic macroinvertebrates collected in 2019 were randomly sampled from riffle habitats with a Surber sampler (0.093 m^2^, 250-µm mesh) using a composite sampling method. Four Surber samples from the site were combined in the field and a quadrate splitting tool was used to randomly select 1/4th of the composite sample for laboratory processing [70]. Samples were preserved in 95% ethanol and transported back to the laboratory. Aquatic insects were enumerated, identified to family level (non-insects to subclass or ordinal levels), and classified into their respective FFGs (collector-gatherers, scraper-grazers, collector-filterers, shredders, or predators; [71]). Each composite sample was sub-sampled (1/16th) for macroinvertebrate enumeration and identification due to high abundance of individuals in each composite sample.

### 2.3. Microplastic Quantification and Characterization

A sub-set of aquatic macroinvertebrates that spanned representative taxa and FFGs were randomly selected from field samples for microplastic quantification in animal tissues (Table 2). Macroinvertebrates were rinsed with lab water for 30 s over a 0.297 mm sieve to externally clean individuals and remove any microplastics externally attached. Macroinvertebrates were individually placed into glass beakers, covered with aluminum foil, and were oven-dehydrated at 75 °C for at least 24 h in preparation for microplastic extraction, similar to McNeish et al. [10]. Dried samples underwent wet peroxide oxidation (20 mL 0.05 M Fe(II) and 20 mL 30% H_2_O_2_ [Fisher Scientific, Hampton, NH, USA]) to digest macroinvertebrate tissues without impacting the integrity of microplastic particles [10,72]. If organic matter was still visible, an additional 20 mL of 30% H_2_O_2_ was added until all organic material was digested or until a total maximum of five additions of 30% H_2_O_2_ (100 mL total) had been added to the sample. The digested samples were each filtered onto 0.7 µm or 0.8 µm gridded filters (Whatman^®^ glass fiber filters or Advantec^®^ cellulose membrane filters, respectively [Fisher Scientific, Hampton, NH, USA]). Filters were examined for microplastics via light microscopy at 25–90 × magnification. Microplastics were enumerated and categorized by color and morphology categories (fiber, fragment, bead, foam, or film [73,74]). Each filter was examined at least two separate times to ensure that microplastic counts were consistent and conservative between lab technicians using light microscopy (AmScope Microscopes, Irvine, CA, USA).

Confirmation of microplastic identification was conducted using Rose Bengal (4,5,6,7,-tetrachloro-2′,4′,5,7′-tetraiodofluorescein [Sigma-Aldrich, St. Louis, MO, USA]) as a second line of microplastic verification in conjunction with light microscopy [13]. Rose Bengal dye stains organic material a pink or purple color, leaving inorganic material (e.g., plastics) unstained and easily discernable from natural-based anthropogenic particles (e.g., cellulose fibers; Figure S1). A 10% subset of all sample filters (i.e., 10% of all macroinvertebrate individuals) across sampling years and taxa were stained with 5 mL of 200 mg L^−1^ Rose Bengal dye (dissolved in 0.45 µm filtered DI water) for 5 min, rinsed with filtered water on a vacuum filtration apparatus, and transferred to Petri slides for microplastic enumeration and categorization by color and morphology as explained previously for macroinvertebrate samples. We compared mean microplastic counts between the light microscopy and Rose Bengal methods and calculated the percent error for microplastics counted on sample filters with the light microscopy method only ([(microscope—Rose Bengal)/Rose Bengal] × 100). In addition, a subset of the largest and smallest fibers (only morphology detected) was mounted onto microscope slides with a rosin liquid adhesive, photographed at 50 to 90× magnification, and each particle’s length and width was measured with the Image J program to confirm our minimum detection limit of particle length at 125 µm (Figure S3). These fibers included a mixture of particles that were not stained (microplastics) and were stained (natural-based anthropogenic particles) after exposure to the Rose Bengal dye. This subset of fibers was analyzed for material type (e.g., plastic polymer, cellulose) with a PerkinElmer Spotlight 200i µ-Fourier transform infrared spectroscopy (ATR mode [attenuated total reflectance] with 60% or greater reference material library match [PerkinElmer, Waltham, MA, USA]) to further confirm our effectiveness of using Rose Bengal to differentiate between microplastic and natural-based anthropogenic particles (Figure S1).

### 2.4. Laboratory Microplastic Contamination Quantification

Laboratory controls were conducted to account for any laboratory microplastic contamination in aquatic macroinvertebrate samples. Clean beakers with no field samples underwent wet peroxide oxidation digestion (20 mL 0.05 M Fe(II) and 20 mL 30% H_2_O_2_) similar to macroinvertebrate samples (*n* = 10 laboratory controls). Laboratory controls were then vacuum filtered on to 0.8 µm filters (Advantec^®^ cellulose membrane filters) and stored in aluminum foil-covered weigh boats. All laboratory control filters were processed for microplastics with both the light microscopy and Rose Bengal methods as previously explained for macroinvertebrate samples. The mean microplastic contamination in laboratory digestion controls (mean No. filter^−1^) was 1.7 (±0.4 SE) microplastic fibers. The rounded mean microplastic fiber contamination (i.e., two whole fibers) was subtracted from each filter’s original stereomicroscopy count and corresponding color category that matched the frequency of contaminated fiber colors. The corrected microplastic abundance values were used to calculate microplastic concentration (No. Macroinvertebrate^−1^) for each taxon, FFG, and site [10]. We also compared the abundance, morphology, and color patterns of laboratory contamination fibers to fibers present in aquatic macroinvertebrate samples.

### 2.5. Statistical Analysis

Generalized linear mixed models (GLMMs) were used to determine if microplastic abundance (No. Macroinvertebrate^−1^) patterns in aquatic macroinvertebrates were associated with time, percentage LULC (forest, urban, and agriculture), and dominant LULC category in watersheds and macroinvertebrate FFG [75,76,77]. The best statistical distribution (Gaussian, Poisson, Zero-inflated negative binomial [ZINB], Zero-inflated Poisson [ZIP], or Negative binomial [NB]) for this pooled dataset was identified as NB with model selection (model.sel() [MuMIn package Version 1.43.17]; [78]) and Akaike’s information criterion corrected for sample size (AIC_c_; Table S1). A series of NB GLMM analyses (glmmTMB(), [glmmTMB package Version 1.1.14]; [79]) were constructed with stream site as a random effect and all other variables as fixed effects. Year was converted to a numerical dummy variable for all models. Continuous variables were checked for autocorrelation (cor() [stats package Version 3.6.2]; [80]). No models were constructed with percent agricultural land use in combination with either percent forest or percent urban land use due to autocorrelation (*r* ≥ |0.3|). All univariate and multivariate model possible combinations were explored with and without interaction parameters (34 models total + Null model) and the best fitting and competing univariate, interaction, and additive models were chosen for final model selection. The overall best model was determined by ranking models based on model weights (*w_i_*) and AIC_c_ (Table 3). Model residuals extracted (simulateResiduals() [DHARMa Version 0.4.3]; [81]) from the best fitting model were found to have no outliers with no significant dispersion and did not significantly deviate from uniformity. The 95% confidence interval was calculated for model variables for the best fitting model (confint() [stats package Version 3.6.2]; [80]). Competing models with the best fitting model were identified within an AICc difference (ΔAICc) of 2 from the top-performing model. Models were excluded from model selection analyses when model convergence and non-positive-definite Hessian matrix issues could not be resolved.

Similar GLMM analyses were conducted for data sub-sets at the family-taxonomic level and for each FFG to determine if time and percent forest, urban, and agricultural land use in watersheds explained microplastic abundance patterns observed for each taxon and FFG. A total of four taxa (out of 36 taxa total) had a large enough sample size (Chironomidae, Elmidae, Hirudinea, and Hydropsychidae) across sites and years sampled for GLMM statistical analyses (Table S2). Diagnostics for all taxonomic and FFG best fitting models revealed no concerns regarding uniformity, dispersion, and outliers for model residuals. In cases when the Null model was a competing model to a taxonomic family’s or FFG’s best fitting model, then the best fitting model was compared to the Null model via loglink ratio to determine if there was a significant difference between models (anova() [stats package Version 3.6.2] and lrtest() [lmtest package Version 0.9-40]; [82]) to increase confidence in the best fitting model. Multivariate models were checked for collinearity: all variables had a variation inflation factor < 5 and were considered to not be collinear (check_collinearity() [performance package Version 0.9.0]; [83]).

Generalized linear models (GLMs) were conducted to determine if microplastic abundance in aquatic macroinvertebrates were influenced by stream site and year sampled, similar to GLMM analyses. Diagnostics for the best fitting model revealed no concerns regarding uniformity, dispersion, and outliers for model residuals. The Null model was not a competing model.

Microplastic abundance was also compared among aquatic Chironomidae life stages to address our prediction that all life stages would have microplastics in their tissues. The Chironomidae family was the only taxonomic group with enough representative individuals for both larva (*N* = 29 indv.) and pupa (*N* = 17 indv.) for the 1998 and 2019 sampling years; therefore, no other life stage comparisons were conducted for other taxonomic groups. Chironomidae microplastic abundance data were non-normal (Shapiro–Wilk test: shapiro.test() [stats package Version 3.6.2]; [80]) and were compared between larva and pupa life stages with Wilcoxon Signed Rank test (wilcox.test() [stats package Version 3.6.2]; [80]) for the 1998 and 2019 sampling years.

We compared microplastic color frequencies for each site within each year sampled with a Chi-square test of independence to determine if there was an association between categories and field sites (chisq.test() [stats package Version 3.6.2]; [80]). Laboratory controls were also included in these analyses to discern if microplastic categories from environmental samples were unique from microplastic contamination in laboratory controls. In addition, microplastic abundance between visual light microscopy and the Rose Bengal methods was conducted with a paired Wilcoxon signed rank test after data were found to not be normally distributed. Microplastic morphology categories were not analyzed since all particles were classified as plastic fibers. All statistical analyses were conducted in R Version 4.1.1 “Kick Things” [84] in the ‘base’ package unless previously stated otherwise.

## 3. Results

### 3.1. Comparison of Microplastic Abundance Among Macroinvertebrate Taxa and Life Stages

A total of 411 macroinvertebrates across 36 taxa were examined for microplastics from six headwater streams (Table 2). We observed microplastic particles only in 134 (approximately 32.6%) individuals from 24 (approximately 66.7%) macroinvertebrate taxa across sampling years and sites, with microplastic abundance in individuals varying from zero to six particles per individual (Table S2). The ANOVA Model III analysis did not reveal significant microplastic patterns among macroinvertebrate taxa (*X*^2^_[35]_ = 12.268, *p* > 0.05); however, 18.1% of all 408 microplastics were present in Elmidae larvae, 12.0% in Hydropsychidae larvae, 9.5% in Psephenidae larvae, and 9.3% in Chironomidae larvae (Table S2). Microplastic abundance in macroinvertebrates peaked to a total of 251 particles in 2010 (approximately 47%) and was the lowest in 2019 with a total of 15 particles (5%). All non-insect taxa, except for Corbiculidae and Planariidae, had either zero or one microplastic particle among all individuals (Table S2).

Microplastic abundance was similar between Chironomidae larvae and pupae for the 1998 and 2010 sampling years (Wilcoxon Test: 1998: W = 68, *p*-value = 0.1324; 2010: W = 62, *p*-value = 0.4329). Microplastics were present in both life stages and greater in larvae (1.79 ± 0.76 SEM) compared to pupae (0.29 ± 0.18 SEM) in 1998. In contrast, mean microplastic abundance was lower in larvae (0.87 ± 0.39 SEM) compared to pupae (2.9 ± 1.88 SEM) in 2010.

### 3.2. Comparison of Microplastic Abundance Among Different Functional Feeding Groups

Microplastics were present in all FFGs, with microplastic abundance the greatest in collector-gatherers (145 total particles [35.6%]) and the least in shredders (21 total particles [5.1%]; Table S3). Macroinvertebrate FFGs were not a significant variable explaining microplastic patterns (*X*^2^_[35]_ = 12.268, *p* > 0.05). Similar to taxonomic patterns, microplastic abundances tended to peak in 2010 among FFGs and was the lowest in 2019 (Table S3).

### 3.3. Comparison of Microplastic Abundance Among Different Sites and Land Use Types

**Figure 2:**
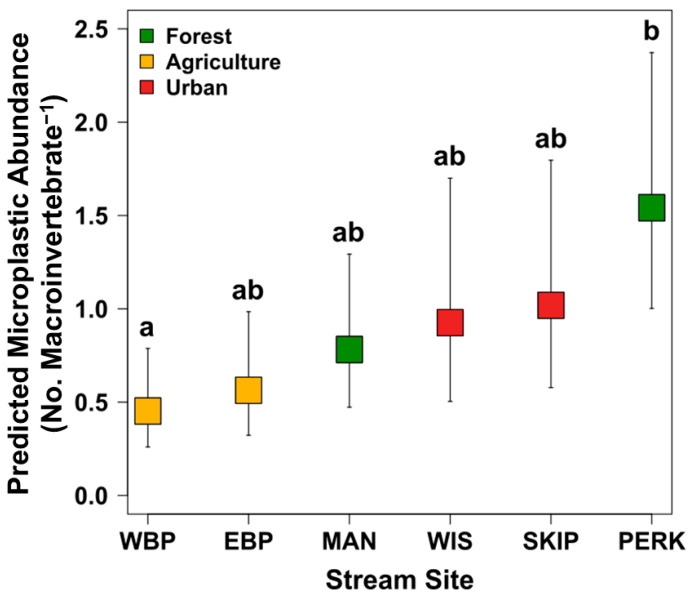
Predicted microplastic abundance (with 95% confidence intervals) for stream sites across sampling years. Data were collected in the Schuylkill River Watershed in southeastern Pennsylvania at the East Branch Perkiomen (EBP), Manatawny (MAN), Perkiomen (PERK), Skippack (SKP), West Branch Perkiomen (WBP), and the Wissahickon (WIS) creeks in the Schuylkill River watershed. Lower case letters refer to lowest (a) to greatest mean microplastic abundance. Different lowercase letters represent significant pairwise comparisons at *p* < 0.05 among sites.

Microplastics were present in aquatic macroinvertebrates across all sites and dominant LULC categories, with 49.8% of all microplastic particles from macroinvertebrates collected from forest dominated sites, 30.1% from urban sites, and 20.0% from agriculture stream sites (Table S4). Both stream site and dominant LULC category were significant predictors of microplastic abundances in macroinvertebrates (Site: *X*^2^_[5]_ = 15.257, *p* = 0.009; LULC: *X*^2^_[2]_ = 9.755, *p* = 0.007). Microplastic abundance was significantly the lowest in the individuals collected from streams surrounded by agricultural land (0.57 ± 0.14 SEM) compared to forest cover (1.22 ± 0.21 SEM) and urban land use (1.18 ± 0.29; all pairwise comparisons *p* < 0.05); however, there was no significant difference between forested and urban dominated streams. Macroinvertebrates from the agriculturally dominated West Branch Perkiomen stream site had the least amount of microplastics, which was significantly different only from macroinvertebrates collected from the Perkiomen (forested) stream site (Figure 2).

### 3.4. Comparison of Microplastic Abundance Among Different Taxa and Different Time Periods

**Figure 3:**
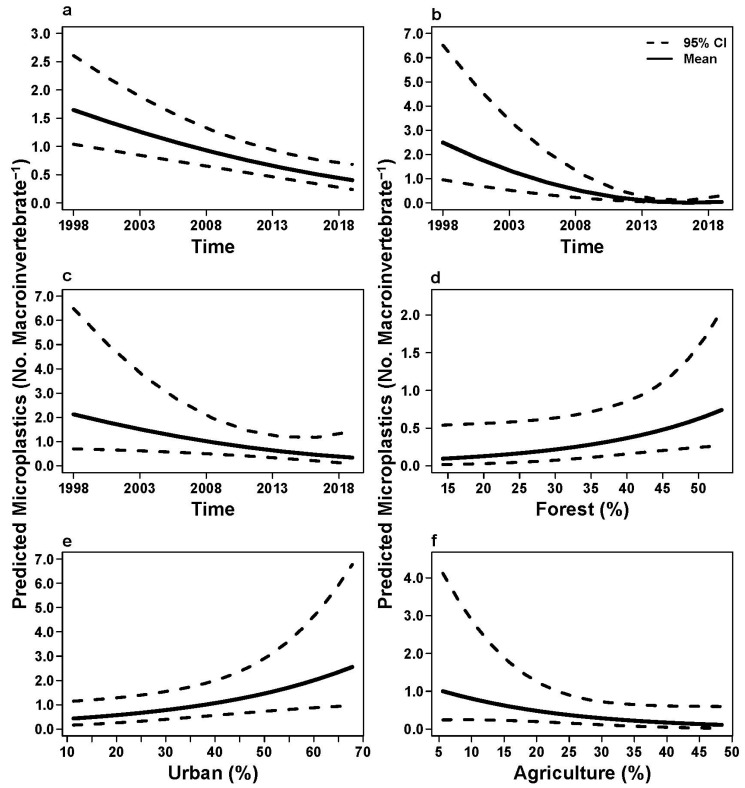
Predicted microplastic abundance (with 95% confidence intervals) for (**a**) pooled, (**b**,**d**,**f**) Chironomidae, and (**c**,**e**) Hydropsychidae macroinvertebrate datasets based on variables from the top generalized linear mixed models. Time represents years; Percent forest, urban, and agriculture represents the percent cover within watersheds. Data were collected in the Schuylkill River Watershed in southeastern Pennsylvania, USA in 1999, 2010, and 2019.

**Figure 4:**
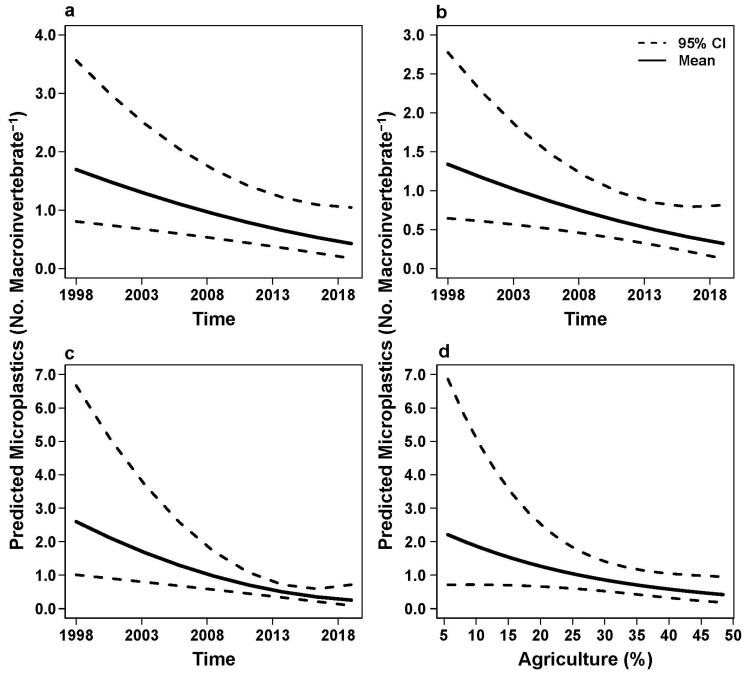
Predicted microplastic abundance (with 95% confidence intervals) for (**a**) collector-gatherer, (**b**) predator, and (**c**,**d**) collector-filterer macroinvertebrate datasets based on variables from the top generalized linear mixed models. Time represents years; Percent agriculture represents the percent cover within watersheds. Data were collected in the Schuylkill River Watershed in southeastern Pennsylvania, USA in 1999, 2010, and 2019.

Statistical models with time as a significant explanatory variable revealed that microplastic abundance decreased through time for the pooled data set and collector-gatherers and predators (Table 4; Figure 2; Figure 3a,b). A significant positive relationship was revealed between microplastic abundance and percent urban land use for Hydropsychidae larvae with time a non-significant variable in the model (Table 4; Figure 3e). Collector-filterer microplastic abundance significantly decreased across time (Table 4; Figure 4c) but had a general negative trend in microplastic abundance with an increase in percent agriculture (Table 4; Figure 4d). Elmidae larvae were the only taxon with the Null model (no explanatory variables) as the best fitted model for microplastic abundance patterns; however, model analysis suggested that microplastic abundance in Hirudinea and Scraper-Grazer and Shredder FFGs were not linked with explanatory variables because the Null models were competing with and were not significantly different from the best fitted models (all *p* > 0.05).

Microplastic abundance in Chironomidae larvae was linked with time and percent forest and agriculture in the landscape, suggesting a potential positive trend in microplastics with an increase in percent forest cover and a decrease in microplastics with a decrease in percent agriculture and across time; however, time was the only significant explanatory variable in the model (Table 4; Figure 3d,f).

Generalized linear mixed models revealed that time and percent forest and agriculture were consistent explanatory variables across best-fitted and competing models (Table 3). Models that contained time as one of the explanatory variables were the most common best fitted models across pooled, taxonomic, and FFG datasets, with 25.21% to 53.09% of the model weights explained by these best fitted models across datasets (Table 3). Best fitting models that had percent forest, agriculture, or urban variables were typically multivariate models that included time as an explanatory variable (Table 3). The FFG variable in multivariate models for the pooled dataset did not meaningfully contribute to neither best-fitted nor competing models.

### 3.5. Comparison of Microplastic Color and Morphology Patterns in Laboratory Controls vs. Preserved Samples

There were significant differences among sites in microplastic colors collected within a year (1998: *χ^2^* = 94.329, *df* = 30, *p*-value < 0.0001; 2010: *χ^2^* = 83.766, *df* = 24, *p*-value < 0.0001; 2019: *χ^2^* = 75.079, *df* = 42, *p*-value = 0.0013). Blue, white, and clear fibers were the most abundant across sites during 1998 and 2019, while white and clear fibers were the predominant fibers across sites in 2010 (Table S5; Figure S2). While laboratory controls were also dominated by blue, white, and clear fibers, these controls were distinct from environmental samples, especially since red fibers were unique to laboratory controls (Table S5; Figure S2). Fibers from 2019 macroinvertebrate samples had the most diverse colors and included pink and yellow colors that were not present in macroinvertebrates from other years (Table S5; Figure S2).

### 3.6. Comparison of Microplastic Abundance in Rose Bengal Dyed vs. Non-Dyed Preserved Samples

Microplastic counts with the light microscopy technique were confirmed using Rose Bengal dye on 10% of samples to confirm that the visual counts of microplastics used for statistical analyses were correctly identified as plastic. We found a significant difference in microplastic counts between methods (V-stat = 58, *p* = 0.0038), with light microscopy microplastic counts significantly less (13 fibers total) than counts recorded from the Rose Bengal method (36 fibers total) across the verified samples. Microplastics identified via light microscopy were approximately 17% (±6 SEM) less than the microplastics identified with the Rose Bengal method, indicating that the microplastic data analyzed for this study (based on light microscopy counts) represented conservative estimates for microplastic presence in aquatic macroinvertebrates across sites, years sampled, taxa, FFG, and percent forest, urban, and agriculture in the landscape.

## 4. Discussion

The ubiquitous nature of plastic debris and the threats in the marine environment, detrimental effects on organisms, prevalence in marine food webs, and overall disruption of marine ecosystems [22,43,44,85,86,87,88,89,90] has been well documented; however, reviews by Wagner et al. [91] and Eerkes-Medrano et al. [92] argued that microplastics are contaminants of emerging concern in freshwater systems and provided key points for future research directions on the presence, distribution, and impact of microplastic pollution on freshwater ecosystems. To date, microplastic pollution has been found within the tissues of cladocerans [93], annelids, crustacean, ostracods, gastropods [94], aquatic insects [95,96,97], and fish [10]. An understanding of microplastic abundance among taxa connected with species traits (life stages and feeding roles) as well as the temporal patterns linked with land use is critical to developing a predictive framework for explaining microplastic abundance in the environment and interactions with aquatic biota.

In this study, we evaluated a unique approach to mine historic data of microplastic presence and abundance through archived macroinvertebrate samples from an on-going water quality study by Stroud Water Research Center in the Schuylkill River Watershed in southeastern Pennsylvania. Relative to our first objective, our findings suggested (depending on the taxon) that microplastic particles were present in Chironomidae larvae and pupae (*P*_1_) with abundance patterns different among years (*P*_4_). The ontogenetic transference of microplastics has been previously documented in mosquitos (Diptera; Culicidae) and drone flies (Diptera: Syrphidae), with microplastic retained in the gut while larva metamorphosed to pupae [98,99,100]. If microplastics are further transferred to adult life stages, then it is possible that aquatic insects may be vectors for the movement of microplastics across ecosystem boundaries. For objective two (i.e., microplastic abundance among functional feeding groups), similar to those findings by Bertoll et al. [101], we found that microplastic abundance was not impacted by all FFGs, lower trophic levels (collector-gatherers/collector filterers and in our study, shredders) tended to have greater microplastic abundances compared to higher trophic levels (predators, refuting *P*_2_). We are not suggesting that there is a preference or selection for microplastics between these FFGs, rather, they are simply ingested through the facilitation of their feeding mode e.g., scraping biofilm and ingesting microplastics trapped within this matrix of organic and inorganic substances within the biofilm or filtering MPs from the water column via collecting/gathering behaviors. The fact that macroinvertebrate gut content analysis studies have shown that they ingest particles from bacteria to macroinvertebrates would suggest that ingestion of microplastics is not constrained by microplastic size [102,103]. These findings are somewhat similar to those by Bertoll et al. [101], except our study suggests that aquatic invertebrates might be exposed to anthropogenic particles from non-food web sources. In addition, microplastic abundances in macroinvertebrates varied with percent land use (objectives 3 and 4) in the landscape and among years (partially supported *P*_3,4_). Findings also suggested that while all microplastic particles were fibers, there were differences in the frequency of fiber colors across sites and years. This distinction may have simply been due to the prevalence of that color in the environment, ease of observation, or possibly due to larvae mistaking the fiber color for a food item [104]. Finally, approximately 17% more microplastics were detected in samples processed with the Rose Bengal dye method compared to samples that were not dyed (supported *P*_5_), suggesting that the microplastic counts reported in this study are underestimated. While beyond the scope of this study, more research is needed to specifically determine connections between microplastic particle traits and the feeding preferences of macroinvertebrates.

Recently, Hou et al. [75] provided a historical context for the pattern of microplastics in fish over more than a century time scale. To our knowledge, though our study covered a three-decade time frame, it provides a similar historical context in aquatic macroinvertebrate microplastic presence that might explain temporal patterns of microplastic concentration in aquatic macroinvertebrates within streams flowing through different land use types over three decades. The Hou et al. [75] study found two distinct periods of with/without microplastics present in fish museum specimens, with a general overall increase in microplastics in fish specimens that they attributed to human population increases and the increase in plastic use and disposal from 1950 to 2018. While we did find an increase in microplastic abundance from archived macroinvertebrates between 1998 and 2010, we also observed a decrease between 2010 and 2019 even though the number of macroinvertebrates processed was similar to other years. Moreover, our models revealed a microplastic decline, overall. This decline could be explained if findings from the water quality study performed by Stroud Water Research Center recommended best management practices to be put in place within specific areas of the Schuylkill River watershed. Another explanation for this decline may be the egestion differences of microplastics among aquatic macroinvertebrates, e.g., while it is known that fish have demonstrated that certain microplastics were egested within 72 h [105], it is unknown what microplastic retention time is for aquatic macroinvertebrates. It is also possible that particle retention within stream sites varied across years, possibly changing microplastic abundance in stream habitats available for interactions with macroinvertebrates. Although this study examined three snapshot years collected over a period of 22 years, it is unknown what the microplastic abundances and concentrations were prior to 1998; however, it might be interpreted that the change of land and water use since colonial times and the advent and increased use of plastic might suggest significant increases longer than observed in this study [75]. More work is needed to develop a predictive framework for understanding temporal and spatial impacts on organismal–microplastic interactions.

For the past 350 years, the history of the Schuylkill River watershed has been intimately tied to the socioeconomic, cultural, and industrial development in the greater Philadelphia, USA, area [67]. A present-day characterization of the Schuylkill River basin would resemble very little of the area from colonial times. Currently, forests have regrown to represent 41% coverage, agriculture covers approximately 40%, and 13% is classified as developed or urban land, in addition to the watershed providing drinking water for more than three million people in this region [67]. Because of these vast changes within this watershed, the question of whether land and water use change has had any effect on microplastic presence remains to be determined within the sampling scope of this study. That said, we did find that microplastics were present in aquatic macroinvertebrates across all sites, but least in the streams within agricultural landscapes. In agricultural systems, subsurface drainage is the most common drainage system in temperate zones [106], but due to the transport of microplastics in soils, microplastics dispersed at the soil surface may still reach deeper soil sections, but over longer periods of time, and eventually reaching the groundwater table [107,108,109,110]. These transport pathways enable microplastics to be transported to the subsurface drainage system and be directly drained to adjacent surface waters [110]. However, the residence and transport times of microplastic in agricultural systems may slow the entry into stream systems [110]. There could be a link between percent agriculture and the amount in the water and sediment habitats but not the macroinvertebrates since the macroinvertebrates were ingesting and egesting particles by selectively feeding. If the use of plasticulture or biosolids from WWTP sludge has ceased or has declined in this watershed, then the absences of these practices could explain why percent agriculture generally was not linked with an increase in microplastics in aquatic macroinvertebrates. For example, WWTPs can be significant sources of microplastics [111], and considering that there are approximately 216 WWTPs located within the Schuylkill River watershed, WWTPs could be a significant contributor of microplastics in streams within urban systems, but also if they were located upstream of forested regions of the watershed. The aforementioned changes in land and water uses in the Schuylkill River watershed highlight the need for future microplastic research to span local and regional spatial scales, especially since proximity to microplastic sources might have stronger effects on microplastic–organismal interactions compared to regional sources throughout watersheds, such as with our regional study.

Predictions from GLMM analyses showed that the percentage of microplastics increased over time in both urban and forested landscapes. Specifically, we observed the highest abundance of microplastics within one of the forested streams (Perkiomen), a finding that may be attributed to the aerial or atmospheric movement of microplastics from urban emissions where they originate and may be deposited via wet or dry deposition into forested areas of a watershed [112,113]. This finding underscores the importance of monitoring both dry and wet deposition of microplastics from one location to another especially to evaluate how atmospheric air currents and subsequent deposition may exacerbate microplastic dispersal across landscapes [114]. Furthermore, the anthropogenic contributions of microplastic into streams within an urban land use can enter waterways via a number of sources from runoff of impervious surfaces, incineration facilities, atmospheric fallout, and other pathways [20]. Increases of microplastic presence and abundance within macroinvertebrates (specifically, Hydropsychidae caddisflies) residing in the urban streams of this study, may in fact be due to the surface runoff via roadways [115]. Because Hydropsychidae caddisflies are net-spinners, i.e., constructing silken nets to filter food particles adrift in the currents [116], it is surprising that they were not one of the macroinvertebrates with the highest microplastic abundance in this study; they have been found in other studies to contain significantly more microplastics than other taxa [95]. Possibly, microplastic particles available to macroinvertebrates may have settled out of the water column and been more readily available to collector-gatherers.

When introduced into aquatic environments (freshwater and marine) by stormwater flows, WWTP effluents, or aerial deposition, microplastics potentially pose as a threat to not only aquatic organisms but also terrestrial predators that may prey on aquatic organisms [117]. To date, approximately 700 aquatic species, many of which are marine, have been documented to be impacted and adversely affected through the ingestion of microplastics [118]. Al-Jaibachi et al. [119] suggested that the ingestion of microplastics by freshwater macroinvertebrates poses a threat to both aquatic predators of these insects as well as terrestrial predator counterparts as aquatic taxa emerge for a terrestrial existence. While many aquatic species spend their entire lives in a stream, lake, or pond, terrestrial habitats are required for certain stages of their life cycle (e.g., eggs or adult developmental stages; [120]). Because many benthic macroinvertebrates may spend most of their lives in freshwater habitats, they have the potential to ingest significant amounts of microplastics as documented in a limited fashion within Oligochaeta, Chironomidae (Family: Diptera;), Ephemeroptera (Families: Siphlonuridae, Heptageniidae, and Baetidae), Odonata (Family: Lestidae), and Trichoptera (Family: Hydropsychidae; [95,96,97,121]). Therefore, studies are needed to explore aquatic–terrestrial linkages as adult aquatic insects emerge from freshwater ecosystems to terrestrial ecosystems, possibly serving as a pathway for microplastic movement from aquatic food webs to terrestrial food webs.

Whether they are archived from ongoing research projects or used in museum collections, preserved macroinvertebrate specimens are a potential source of preserved data to determine historical patterns of microplastic pollution. These preserved specimens can be used to address ecological questions such as the impact that land and water use has across the landscape or trophic passage of microplastics into higher organisms, but more importantly, to understand future trends that might dictate how such changes might be impacted by microplastic pollution [75]. Our study documented the presence and abundance of microplastics within eight aquatic insect orders and 29 families as well as seven non-insect orders over a 22-year period (Table 2), in contrast to snapshot studies from a single temporal perspective, for example, Nel et al. [97], who focused on the presence of microplastics in a South African river system; Windsor et al. [95] in Welsh rivers; and the study by Akindele et al. [96], which focused on a Nigerian river study. All these aforementioned studies selected these taxa as bioindicators to address either microplastic pollution levels and/or feeding guild questions and potentially, these studies may have documented the presence of microplastics among other aquatic insect orders/families had their studies been expanded from a survey perspective. In addition, it is important that future studies using archived specimens consider the possible impacts that the preservation method may have when extracting and identifying microplastics. For example, our archived specimens may have been contaminated with microplastics, since these samples pre-date common laboratory practices to filter solutions to remove microplastics before exposure to samples. It is also possible that the preservation solution increased the brittleness of the microplastic particles, potentially resulting in increased particle fragmentation and inflating microplastic abundances detected in this study. Finally, the preservation solutions used for archived specimens may interfere with dye staining methods used to identify microplastics, which might result in underestimating microplastic abundance in archived specimens.

Utilizing archived specimen collections allowed us to demonstrate that not only are microplastics prevalent among a diverse list of pollution tolerant and intolerant taxa, but that microplastic pollution within these taxa has varied over this time period. In this study, we found that collector-gatherers and collector-filterers had the greatest abundance of microplastics; however, predators had intermediate levels of microplastics (Table S3), hinting that microplastics might not be biomagnifying in the aquatic macroinvertebrate part of the food web. Future studies might detect unique microplastic biomagnification and food web patterns if explored at different spatial scales (e.g., site vs. region). While not as temporally extensive as the 100+ year study on the increase of microplastics in fish tissue over time by Hou et al. [75], our study found that microplastic abundance decreased over time and to our knowledge, no prior study has explored historical patterns in aquatic macroinvertebrates in connection to species traits and differences in land use; therefore, results from this study provide novel findings that can inform management efforts on terrestrial–aquatic connections that link microplastic abundance in freshwaters and aquatic biota.

## Figures and Tables

**Table 1:** Site descriptions with dominant and percent (%) watershed land use/landcover features for all streams sampled in this study within the Schuylkill River watershed.

Site	Dominant Land Use	Forest (%)	Urban (%)	Agriculture (%)	Watershed Area (km^2^)	Latitude, Longitude
Perkiomen Creek	Forest	52.7	15.3	30.2	710	40°8′43.9″ N, −75°27′20.9″ W
Manatawny Creek	Forest	53.2	11.3	35.4	180	40°20′14.9″ N, −75°44′26.0″ W
Skippack Creek	Urban	14.3	53.6	32.1	51	40°9′2.9″ N, −75°26′47.0″ W
Wissahickon Creek	Urban	25.7	67.8	5.6	146	40°4′41.9″ W −75°13′29.9″ N
East Branch Perkiomen Creek	Agriculture	28.1	33.4	38.4	157	40°17′53.0″ N, −75°25′12″ W
West Branch Perkiomen Creek	Agriculture	38.4	12.9	48.4	62	40°23′45.8″ N, −75°36′30.8″ W

**Table 2:** Summary of information about the aquatic macroinvertebrates (i.e., aquatic insects plus non-insects) used in this study. A cumulative number of larvae and pupae (in parentheses) is shown for insects, and for non-insects the number of individuals is presented. FFGs represents macroinvertebrate functional feeding groups: collector-filterers (CF), collector-gatherers (CG), predators (PR), scraper-grazers (SG), and shredders (SH). Water body study sites included the East Branch Perkiomen (EBP), Manatawny (MAN), Perkiomen (PERK), Skippack (SKP), West Branch Perkiomen (WBP), and the Wissahickon (WIS) creeks in the Schuylkill River watershed. The dominant watershed land use/land cover (LULC) included forest (F), urban (U), and agriculture (A).

Order	Family	No. Indv.	FFGs	Water Body	Years	LULC
Coleoptera	Elmidae	44	CG	EBP, MAN, PERK, SKP, WBP, WIS	1998, 2010, 2019	F, U, A
Coleoptera	Psephenidae	29	SG	ESP, MAN, PERK, SKP, WBP	1998, 2010, 2019	F, U, A
Diptera	Chironomidae	47 (17)	CG	EBP, MAN, PERK, SKP, WBP, WIS	1998, 2010, 2019	F, U, A
Diptera	Simuliidae	20	CF	MAN, PERK, SKP, WBP, WIS	1998, 2010, 2019	F, U, A
Diptera	Tipulidae	17	SH	MAN, PERK, WBP, WIS	1998, 2010, 2019	F, U, A
Diptera	Empididae	14	PR	WBP, MAN, PERK, WBP, WIS	1998, 2010, 2019	F, U, A
Diptera	Limoniidae (Tipulidae: Subfamily)	2	SH	WIS	2019	U
Diptera	Athericidae	1	PR	MAN	2019	F
Ephemeroptera	Ephemerellidae	26	CG	EBP, MAN, PERK, SKP, WBP, WIS	1998, 2010, 2019	F, U, A
Ephemeroptera	Heptageniidae	15	SG	EBP, MAN, PERK, WBP	1998, 2010, 2019	F, A
Ephemeroptera	Baetidae	6	CG	PERK, WBP	2010, 2019	F, A
Ephemeroptera	Leptophlebiidae	5	CG	PERK, WBP	1998, 2010	F, A
Lepidoptera	Crambidae	5	SH	EBP, PERK, SKP	1998, 2010, 2019	F, U, A
Neuroptera	Sisyridae	1	PR	WIS	2019	U
Odonata	Coenagrionidae	2	PR	EBP, SKP	1998, 2010	U, A
Plecoptera	Perlidae	8	PR	MAN, PERK, WBP	1998, 2010, 2019	F, A
Plecoptera	Capniidae	7	SH	EBP, MAN, SKP, WBP	1998, 2010, 2019	F, U, A
Plecoptera	Nemouridae	2	SH	EBP	1998	A
Plecoptera	Perlodidae	1	PR	SKP	2019	U
Trichoptera	Hydropsychidae	38	CF	EBP, MAN, PERK, SKP, WBP, WIS	1998, 2010, 2019	F, U, A
Trichoptera	Philopotamidae	21	CF	EBP, MAN, PERK, SKP, WBP, WIS	1998, 2010, 2019	F, U, A
Trichoptera	Hydroptilidae	12	SG	EBP, MAN, PERK, SKP, WBP, WIS	1998, 2010, 2019	F, U, A
Trichoptera	Helicopsychidae	3	SG	MAN, PERK, SKP	1998	F, U, A
Trichoptera	Limnephilidae	2	SH	MAN, WBP	2010, 2019	F, A
Trichoptera	Odontoceridae	2	SH	MAN, WBP	2019	F, A
Trichoptera	Uenoidae	2	CG	MAN, PERK	1998	F
Trichoptera	Glossosomatidae	1	SG	SKIP	1998	U
Trichoptera	Goeridae	1	SG	MAN	2019	F
Trichoptera	Rhyachophilidae	1	PR	WBP	2010	A
Clitellata (Class)	Hirundinea (Subclass)	37	PR	EBP, MAN, PERK, SKP, WBP, WIS	1998, 2010, 2019	F, U, A
Acari (Subclass)	Hydrachnidae	21	PR	EBP, MAN, PERK, SKP, WBP, WIS	1998, 2010, 2019	F, U, A
Tricladida	Planariidae	9	PR	EBP, PERK, SKP, WBP, WIS	1998, 2010, 2019	F, U, A
Venerida	Corbiculidae	6	CF	EBP, MAN, PERK, SKP, WBP	1998, 2010, 2019	F, U, A
Amphipoda	Gammaridae	4	SH	EBP, MAN, WBP, WIS	1998, 2010, 2019	F, U, A
Isopoda	Asellidae	1	SH	SKP	2010	U
Unionida	Unionidae	1	CF	PERK	2019	F
	TOTAL =	411				

**Table 3:** Model selection results evaluating the best and competing models for aquatic macroinvertebrate microplastic abundance for the pooled (all taxa) dataset, taxa, and functional feeding groups. Null models were included as reference, regardless if the null were or were not competing models. LL = the log-link ratio; AIC_C_ = Akaike’s information criterion corrected for sample sizes; ΔAIC_C_ = the difference from the best model; *w_i_* = the AIC_C_ weight.

Model	*df*	LL	AICc	ΔAICc	*w_i_*
Pooled (Site fixed effect)					
Site + Year	8	−497.12	1010.59	0.00	0.9902
Null (intercept with no random effects)	2	−512.08	1028.19	17.61	0.0001
Pooled (Site random effect)					
Time	4	−502.61	1013.32	0.00	0.2521
Time + Agriculture (%)	5	−501.72	1013.58	0.26	0.2211
Time + Forest (%) + Agriculture (%)	6	−501.19	1014.58	1.26	0.1341
Time + Forest (%)	5	−502.48	1015.10	1.78	0.1033
Null (intercept with random effects)	3	−510.40	1026.85	13.53	0.0003
Chironomidae					
Time + Agriculture (%) + Forest (%)	6	−43.29	100.68	0.00	0.5028
Time	4	−46.26	101.48	0.80	0.3375
Null (intercept with random effects)	3	−52.25	111.05	10.37	0.0028
Elmidae					
Null (intercept with random effects)	3	−68.89	144.38	0.00	0.6891
Time	4	−68.47	145.97	1.59	0.3109
Hirudinea					
Time	4	−43.32	95.89	0.00	0.3001
Null (intercept with random effects)	3	−44.82	96.38	0.49	0.2349
Time + Agriculture (%)	5	−42.31	96.56	0.67	0.2145
Time × Agriculture (%)	6	−41.18	97.17	1.28	0.1583
Hydropsychidae					
Time + Urban (%)	5	−50.77	113.42	0.00	0.3320
Time + Agriculture (%)	5	−51.57	115.02	1.60	0.1492
Forest (%)	4	−52.98	115.17	1.75	0.1386
Time × Urban (%)	6	−50.35	115.41	1.99	0.1229
Null (intercept with random effects)	3	−54.55	115.80	2.38	0.1010
Scraper-Grazers					
Agriculture (%)	4	−74.51	157.74	0.00	0.5569
Null (intercept with random effects)	3	−76.37	159.17	1.42	0.2733
Collector-Gatherers					
Time	4	−169.17	346.65	0.00	0.2971
Time + Forest (%)	5	−168.85	348.17	1.52	0.1390
Null (intercept with random effects)	3	−171.92	350.03	3.39	0.0546
Collector-Filterers					
Time + Agriculture (%)	5	−102.50	215.76	0.00	0.5309
Time	4	−104.55	217.60	1.85	0.2108
Null (intercept with random effects)	3	−108.00	222.30	6.54	0.0201
Shredders					
Time	4	−44.06	97.33	0.00	0.3975
Null (intercept with random effects)	3	−45.59	97.89	0.56	0.3009
Time + Forest (%)	5	−43.51	98.90	1.57	0.1815
Predators					
Time	4	−104.50	217.45	0.00	0.3174
Time + Agriculture (%)	5	−104.05	218.77	1.32	0.1641
Time + Urban (%)	5	−104.11	218.90	1.45	0.1536
Null (intercept with random effects)	3	−106.62	219.51	2.06	0.1132

**Table 4:** Model coefficients, statistical results, and 95% confidence intervals from the top models evaluating the effects of time and percent forest, urban, and agriculture cover in watersheds on microplastic abundance in macroinvertebrates across pooled data (all taxa), individual taxa, and functional feeding groups. Statistically significant *p*-values (*p*) are underlined. SE = standard error, Z = Z-statistic, 95% CI = 95% confidence interval.

					95% CI	
Coefficient	Estimate	SE	Z	*p*	Lower	Upper
Pooled: Site Random						
Intercept	0.7820	0.3627	2.156	0.0311	0.012	1.599
Site:MAN	0.3275	0.3834	0.854	0.3929	−0.428	1.082
SitePERK	1.0063	0.3593	2.801	0.0051	0.295	1.712
Site:SKIP	0.5920	0.4003	1.479	0.1391	−0.189	1.387
Site:WBP	−0.2199	0.4027	−0.546	0.5851	−1.015	0.572
Site:WIS	0.4963	0.4168	1.191	0.2337	−0.311	1.323
Time	−0.6945	0.1402	−4.953	<0.0001	−1.050	−0.349
Pooled						
Intercept	1.2021	0.3786	3.175	0.0015	−0.460	1.944
Time	−0.7043	0.1802	−3.908	<0.0001	−1.058	−0.351
Chironomidae						
Intercept	2.7286	1.4118	1.933	0.0533	−0.039	5.496
Time	−2.0432	0.6454	−3.166	0.0016	−3.308	−0.778
Forest (%)	0.0531	0.0276	1.924	0.0544	−0.001	0.107
Agriculture (%)	−0.0513	0.0303	−1.690	0.0911	−0.111	0.008
Elmidae						
Intercept	0.1622	0.5819	0.279	0.7800	−0.978	1.303
Hirudinea						
Intercept	1.8151	1.1830	1.534	0.1250	−0.500	4.130
Time	−1.0170	0.6029	−1.687	0.0916	−2.200	0.160
Hydropsychidae						
Intercept	0.6290	1.1330	0.555	0.5790	−1.590	2.850
Time	−0.9186	0.5585	−1.645	0.1000	−2.010	0.180
Urban (%)	0.0313	0.0132	2.365	0.0180	0.005	0.060
Scraper-Grazers						
Intercept	2.7603	1.6511	1.672	0.0946	−0.476	5.996
Agriculture (%)	−0.0817	0.0477	−1.713	0.0867	−0.175	0.012
Collector-Gatherers						
Intercept	1.2155	0.6059	2.006	0.0448	0.028	2.403
Time	−0.6875	0.2926	−2.350	0.0188	−1.261	−0.114
Collector-Filterers						
Intercept	3.4052	1.0982	3.101	0.0019	1.253	5.556
Time	−1.1579	0.4347	−2.663	0.0077	−2.010	−0.306
Agriculture (%)	−0.0387	0.0198	−1.953	0.0508	−0.078	0.0001
Shredders						
Intercept	0.7585	0.8421	0.901	0.3677	−0.892	2.409
Year	−0.7171	0.4202	−1.706	0.0879	−1.541	0.107
Predators						
Intercept	1.0023	0.6746	1.486	0.1373	−0.320	2.324
Year	−0.7102	0.3464	−2.050	0.0404	−1.389	−0.031

## Data Availability

The data presented in this study are available on request from the corresponding author. The data are not publicly available due to privacy.

## Supplementary Materials

The following supporting information can be downloaded at: https://www.mdpi.com/article/10.3390/insects17040386/s1, Table S1: Akaike’s information criterion corrected values for sample sizes (AICc) for the statistical distribution of microplastic abundance (No. Macroinvertebrate-1) for pooled data and macroinvertebrate taxa collected from 1998, 2010, and 2019. Distributions tested included the negative binomial (NB), zero-inflated negative binomial (ZINB), zero-inflated Poisson (ZIP), Poisson, and Gaussian. Table S2: Summary statistics for mean microplastics (MP) present in aquatic macroinvertebrate taxa in 1998, 2010, and 2019 sampling years. *n* = no. individuals, *s* = standard deviation, SEM = standard error of the mean, No. MP = number of minimum and maximum microplastics per individual, Family Total MP = summed microplastics found across years for each family. Table S3: Summary statistics for mean microplastics (MP) present in aquatic macroinvertebrate functional feeding groups (FFG) in 1998, 2010, and 2019 sampling years. *n* = no. individuals, *s* = standard deviation, SEM = standard error of the mean, No. MP = number of minimum and maximum microplastics per individual. Table S4: Summary statistics for mean microplastics (MP) present in aquatic macroinvertebrate among sites’ dominant land use/land cover (LULC) categories in 1998, 2010, and 2019 sampling years. *n* = no. individuals, *s* = standard deviation, SEM = standard error of the mean, No. MP = number of minimum and maximum microplastics per individual. Table S5: Relative abundance (%) of microplastic fiber colors across sites for each year and for laboratory controls. Figure S1: Examples of anthropogenic particles after Rose Bengal dye staining to differentially stain (a) microplastic and (b,c) natural-based anthropogenic particles with associated µ-FTIR spectra. Percent matches reported indicate sample material spectra (black lines) in comparison to reference library spectra (red & green line) matches. Note that the blue cellulose fiber (c) has a light purple tinted color due to the natural-based anthropogenic particle stained with the Rose Bengal over the fiver’s blue pigment. Figure S2: Relative abundance (%) of microplastic fiber color in environmental samples during (a) 1998, (b) 2010, and (c) 2019. Control data represents relative abundance of microplastics fibers from laboratory controls. Figure S3: Microplastic fiber (a) length (µm) and (b) width (µm) distributions across a subset of particles picked from samples collected in 1998 and 2019 (*n* = 18).
