# Supplementary material for: Archived Historical Aquatic Macroinvertebrate Specimens Suggest Connections Between Microplastic Abundance Patterns, Trophic Traits, and Land Use
Source: Insects. 2026 Apr 2;17(4):386. doi: 10.3390/insects17040386 (PMC13115684; doi:10.3390/insects17040386)
Supplement: Supplementary file 1 [file insects-17-00386-s001.zip › insects-4156445-supplementary.pdf]

**Supplementary Materials:**

**Title**: Archived historical aquatic macroinvertebrate specimens suggest connections between microplastic abundance patterns, trophic traits, and land use

**Scientific Journal:** Insects

**Authors Information**: Rachel E. McNeish[1*], Marisa D. Macchia[2], Nicole M. Lee[2], Austin T. Harrison[2], Alexandra J. Brown[1], John K. Jackson[3], and John R. Wallace[2*]

**Affiliations:**
[1]Department of Biology, California State University, Bakersfield, Bakersfield, CA 93311, USA
[2]Department of Biology, Millersville University, Millersville, PA 17551, USA
[3]Stroud Water Research Center, Avondale, PA 19311, USA
*Correspondence: rae.mcneish@gmail.com (R.E.M.); john.wallace@millersville.edu (J.R.W.)

**Table S1:** Akaike's information criterion corrected values for sample sizes (AICc) for the statistical distribution of microplastic abundance (No. Macroinvertebrate-1) for pooled data and macroinvertebrate taxa collected from 1998, 2010, and 2019. Distributions tested included the negative binomial (NB), zero-inflated negative binomial (ZINB), zero-inflated Poisson (ZIP), Poisson, and Gaussian.

| Dataset | Distribution | $AIC_c$ | $w_i$ |
|---|---|---|---|
| Pooled | NB | 1,028.19 | 0.7340 |
| | ZINB | 1,030.22 | 0.2660 |
| | ZIP | 1,227.06 | < 0.0001 |
| | Poisson | 1,638.44 | < 0.0001 |
| | Gaussian | 1,937.98 | < 0.0001 |
| Chironomidae | NB | 108.77 | 0.7151 |
| | ZINB | 110.74 | 0.2663 |
| | ZIP | 116.06 | 0.0186 |
| | Poisson | 157.70 | < 0.0001 |
| | Gaussian | 196.18 | < 0.0001 |
| Elmidae | NB | 142.99 | 0.7602 |
| | ZINB | 145.30 | 0.2398 |
| | ZIP | 194.33 | < 0.0001 |
| | Gaussian | 249.60 | < 0.0001 |
| | Poisson | 265.50 | < 0.0001 |
| Hirudinea | NB | 94.00 | 0.7272 |
| | ZINB | 95.97 | 0.2720 |
| | ZIP | 107.78 | 0.0007 |
| | Poisson | 148.76 | < 0.0001 |
| | Gaussian | 178.06 | < 0.0001 |
| Hydropsychidae | NB | 113.44 | 0.7652 |
| | ZINB | 115.80 | 0.2348 |
| | ZIP | 144.30 | < 0.0001 |
| | Poisson | 183.43 | < 0.0001 |
| | Gaussian | 197.82 | < 0.0001 |
| Scraper-Grazers | NB | 156.95 | 0.7516 |
| | ZINB | 159.17 | 0.2484 |
| | ZIP | 199.56 | < 0.0001 |
| | Poisson | 279.25 | < 0.0001 |
| | Gaussian | 312.49 | < 0.0001 |
| Collector-Gatherers | NB | 348.87 | 0.7401 |
| | ZINB | 350.97 | 0.2599 |
| | ZIP | 427.19 | < 0.0001 |
| | Poisson | 574.47 | < 0.0001 |
| | Gaussian | 652.90 | < 0.0001 |
| Collector-Filterers | NB | 220.15 | 0.7447 |
| | ZINB | 222.29 | 0.2553 |
| | ZIP | 258.38 | < 0.0001 |

| | | | |
|---|---|---|---|
| | Poisson | 362.12 | < 0.0001 |
| | Gaussian | 411.86 | < 0.0001 |
| Shredders | NB | 97.24 | 0.7626 |
| | ZINB | 99.60 | 0.2340 |
| | ZIP | 108.10 | 0.0033 |
| | Poisson | 117.46 | < 0.0001 |
| | Gaussian | 151.06 | < 0.0001 |
| Predators | NB | 217.38 | 0.7195 |
| | ZINB | 219.26 | 0.2805 |
| | ZIP | 237.20 | < 0.0001 |
| | Poisson | 305.38 | < 0.0001 |
| | Gaussian | 396.62 | < 0.0001 |

**Table S2:** Summary statistics for mean microplastics (MP) present in aquatic macroinvertebrate taxa in 1998, 2010, and 2019 sampling years. $n$ = no. individuals, $s$ = standard deviation, SEM = standard error of the mean, No. MP = number of minimum and maximum microplastics per individual, Family Total MP = summed microplastics found across years for each family.

| | | 1998 | | | | | 2010 | | | | | 2019 | | | | | |
|---|---|---|---|---|---|---|---|---|---|---|---|---|---|---|---|---|---|
| **Order** | **Family** | ***n*** | **Mean** | ***s*** | **SEM** | **No. MP** | ***n*** | **Mean** | ***s*** | **SEM** | **No. MP** | ***n*** | **Mean** | ***s*** | **SEM** | **No. MP** | **Family Total MP** |
| Coleoptera | Elmidae | 17 | 1.06 | 1.64 | 13.28 | 0 - 5 | 13 | 3.85 | 6.66 | 5.04 | 0 - 22 | 14 | 0.43 | 1.09 | 13.41 | 0 - 3 | 74 |
| | Psephenidae | 9 | 0.11 | 0.33 | 15.59 | 0 - 1 | 14 | 2.71 | 5.76 | 5.83 | 0 - 21 | 6 | 0.00 | 0.00 | - | 0 | 39 |
| Diptera | Chironomidae | 14 | 1.79 | 2.86 | 8.28 | 0 - 10 | 15 | 0.87 | 1.51 | 12.22 | 0 - 5 | 18 | 0.00 | 0.00 | - | 0 | 38 |
| | Tipulidae | 6 | 1.67 | 1.86 | 4.40 | 0 - 5 | 8 | 1.88 | 3.00 | 4.62 | 0 - 9 | 3 | 0.33 | 0.58 | 3.95 | 0 - 1 | 26 |
| | Simuliidae | 1 | 3.00 | | | 3 | 7 | 1.86 | 2.67 | 4.28 | 0 - 7 | 12 | 0.08 | 0.29 | 22.33 | 0 - 1 | 17 |
| | Empididae | 6 | 1.00 | 1.26 | 5.33 | 0 - 3 | 5 | 0.80 | 1.30 | 4.38 | 0 - 3 | 3 | 0.33 | 0.58 | 3.95 | 0 - 1 | 11 |
| | Athericidae | 0 | - | - | - | - | 0 | - | - | - | - | 1 | 0.00 | - | - | 0 | 0 |
| | Limoniidae | 0 | - | - | - | - | 0 | - | - | - | - | 2 | 0.00 | 0.00 | - | 0 | 0 |
| Trichoptera | Hydropsychidae | 13 | 1.08 | 1.38 | 11.06 | 0 - 5 | 13 | 2.69 | 4.94 | 5.85 | 0 - 18 | 12 | 0.00 | 0.00 | - | 0 | 49 |
| | Hydroptilidae | 5 | 1.60 | 2.61 | 3.10 | 0 - 6 | 6 | 1.50 | 1.38 | 5.11 | 0 - 3 | 1 | 0.00 | - | - | 0 | 17 |
| | Philopotamidae | 9 | 0.33 | 0.71 | 10.70 | 0 - 2 | 7 | 1.86 | 4.10 | 3.46 | 0 - 11 | 5 | 0.00 | 0.00 | - | 0 | 16 |
| | Rhyachophilidae | 0 | - | - | - | - | 1 | 1.00 | - | - | 1 | - | - | - | - | - | 1 |
| | Uenoidae | 2 | 0.50 | 0.71 | 2.38 | 0 - 1 | 0 | - | - | - | - | 0 | - | - | - | - | 1 |
| | Glossosomatidae | 1 | 0.00 | - | - | 0 | 0 | - | - | - | - | 0 | - | - | - | - | 0 |
| | Goeridae | 0 | - | - | - | - | 0 | - | - | - | - | 1 | 0.00 | - | - | 0 | 0 |
| | Helicopsychidae | 3 | 0.00 | 0.00 | - | 0 | 0 | - | - | - | - | 0 | - | - | - | - | 0 |
| | Limnephilidae | 0 | - | - | - | - | 1 | 1 | - | - | 1 | 1 | 0.00 | - | - | 0 | 0 |
| | Odontoceridae | 0 | - | - | - | - | 0 | - | - | - | - | 2 | 0.00 | 0.00 | - | 0 | 0 |
| | Unionidae | 0 | - | - | - | - | 0 | - | - | - | - | 1 | 0.00 | - | - | 0 | 0 |
| Ephemeroptera | Ephemerellidae | 11 | 1.09 | 1.87 | 8.05 | 0 - 6 | 9 | 1.11 | 2.98 | 5.22 | 0 - 9 | 6 | 0.67 | 1.21 | 5.45 | 0 - 3 | 26 |
| | Heptageniidae | 6 | 0.83 | 1.60 | 4.74 | 0 - 4 | 2 | 3.00 | 0.00 | - | 3 | 7 | 0.00 | 0.00 | - | 0 | 11 |

| | | | | | | | | | | | | | | | | | |
|---|---|---|---|---|---|---|---|---|---|---|---|---|---|---|---|---|---|
| | Leptophlebiidae | 1 | 0.00 | - | - | 0 | 4 | 1.00 | 1.41 | 3.36 | 0 - 3 | - | - | - | - | - | 4 |
| | Baetidae | 0 | - | - | - | - | 4 | 0.25 | 0.50 | 5.66 | 0 - 1 | 2 | 0.00 | 0.00 | - | 0 | 1 |
| Arhynchobdellida | Hirudinea | 12 | 1.25 | 1.22 | 10.88 | 0 - 3 | 10 | 1.90 | 4.68 | 4.62 | 0 - 15 | 15 | 0.13 | 0.52 | 20.87 | 0 - 2 | 36 |
| Trombidiformes | Hydrachnidae | 11 | 0.09 | 0.30 | 20.03 | 0 - 1 | 6 | 2.33 | 2.94 | 3.50 | 0 - 7 | 4 | 0.00 | 0.00 | - | 0 | 15 |
| Venerida | Corbiculidae | 2 | 4.00 | 2.83 | 1.19 | 2 - 6 | 3 | 0.33 | 0.58 | 3.95 | 0 - 1 | 1 | 0.00 | - | - | 0 | 9 |
| Plecoptera | Perlidae | 2 | 2.00 | 2.83 | 1.19 | 0 - 4 | 1 | 0.00 | - | - | 0 - 0 | 5 | 0.00 | 0.00 | - | 0 | 4 |
| | Capniidae | 4 | 0.25 | 0.50 | 5.66 | 0 - 1 | 2 | 1.00 | 0.00 | - | 1 | 1 | 0.00 | - | - | 0 | 3 |
| | Nemouridae | 2 | 0.00 | 0.00 | - | 0 | 0 | - | - | - | - | 0 | - | - | - | - | 0 |
| | Perlodidae | 0 | - | - | - | - | 0 | - | - | - | - | 1 | 0.00 | - | - | 0 | 0 |
| Tricladida | Planariidae | 6 | 1.00 | 1.10 | 5.73 | 0 - 2 | 2 | 0.50 | 0.71 | 2.38 | 0 - 1 | 1 | 0.00 | - | - | 0 | 7 |
| Lepidoptera | Crambidae | 2 | 0.50 | 0.71 | 2.38 | 0 - 1 | 2 | 0.00 | 0.00 | - | 0 | 1 | 0.00 | - | - | 0 | 1 |
| Amphipoda | Gammaridae | 2 | 0.00 | 0.00 | - | 0 | 1 | 1.00 | - | - | 1 | 1 | 0.00 | - | - | 0 | 1 |
| Isopoda | Asellidae | 0 | - | - | - | - | 1 | 1.00 | - | - | 1 | - | - | - | - | - | 1 |
| Odonata | Coenagrionidae | 1 | 0.00 | - | - | 0 | 1 | 0.00 | - | - | 0 | 0 | - | - | - | - | 0 |
| Neuroptera | Sisyridae | 0 | - | - | - | - | 0 | - | - | - | - | 1 | 0.00 | - | - | 0 | 0 |

**Table S3:** Summary statistics for mean microplastics (MP) present in aquatic macroinvertebrate functional feeding groups (FFG) in 1998, 2010, and 2019 sampling years. *n* = no. individuals, *s* = standard deviation, SEM = standard error of the mean, No. MP = number of minimum and maximum microplastics per individual

| | 1998 | | | | | 2010 | | | | | 2019 | | | | | |
|---|---|---|---|---|---|---|---|---|---|---|---|---|---|---|---|---|
| **FFG** | ***n*** | **Mean** | ***s*** | **SEM** | **No. MP** | ***n*** | **Mean** | ***s*** | **SEM** | **No. MP** | ***n*** | **Mean** | ***s*** | **SEM** | **No. MP** | **Total MP** |
| Collector-Gatherer | 48 | 1.17 | 2.0 | 0.30 | 0 - 10 | 46 | 1.72 | 4.01 | 0.59 | 0 - 22 | 40 | 0.25 | 0.81 | 0.13 | 0 - 3 | 145 |
| Collector-Filterer | 25 | 1.12 | 1.6 | 0.32 | 0 - 6 | 30 | 2.07 | 3.95 | 0.72 | 0 - 18 | 31 | 0.03 | 0.18 | 0.03 | 0 - 1 | 91 |
| Predator | 38 | 0.84 | 1.2 | 0.19 | 0 - 4 | 26 | 1.50 | 3.24 | 0.64 | 0 - 15 | 31 | 0.10 | 0.40 | 0.07 | 0 - 2 | 74 |
| Scraper-Grazer | 24 | 0.58 | 1.5 | 0.30 | 0 - 6 | 22 | 2.41 | 4.62 | 0.98 | 0 - 21 | 15 | 0.00 | 0.00 | 0.00 | 0 - 0 | 67 |
| Shredder | 13 | 0.92 | 1.4 | 0.40 | 0 - 5 | 14 | 1.29 | 2.33 | 0.62 | 0 - 9 | 11 | 0.09 | 0.30 | 0.09 | 0 - 1 | 31 |

**Table S4:** Summary statistics for mean microplastics (MP) present in aquatic macroinvertebrate among sites' dominant land use/land cover (LULC) categories in 1998, 2010, and 2019 sampling years. *n* = no. individuals, *s* = standard deviation, SEM = standard error of the mean, No. MP = number of minimum and maximum microplastics per individual

| | 1998 | | | | | 2010 | | | | | 2019 | | | | | |
|---|---|---|---|---|---|---|---|---|---|---|---|---|---|---|---|---|
| **LULC** | ***n*** | **Mean** | ***s*** | **SEM** | **No. MP** | ***n*** | **Mean** | ***s*** | **SEM** | **No. MP** | ***n*** | **Mean** | ***s*** | **SEM** | **No. MP** | **Total MP** |
| Forest | 49 | 1.04 | 1.50 | 0.21 | 0 - 6 | 61 | 2.33 | 3.93 | 0.50 | 0 - 21 | 56 | 0.18 | 0.69 | 0.09 | 0 - 3 | 203 |
| Urban | 54 | 0.98 | 1.46 | 0.20 | 0 - 6 | 23 | 3.04 | 5.63 | 1.17 | 0 - 22 | 27 | 0.00 | 0.00 | 0.00 | 0 | 123 |
| Agriculture | 45 | 0.84 | 1.95 | 0.29 | 0 - 10 | 54 | 0.72 | 2.11 | 0.29 | 0 - 15 | 45 | 0.11 | 0.38 | 0.06 | 0 - 2 | 82 |

**Table S5:** Relative abundance (%) of microplastic fiber colors across sites for each year and for laboratory controls.

| Year | Site | Clear or White | Blue | Blue & Clear | Gray | Black | Red | Yellow | Pink |
|---|---|---|---|---|---|---|---|---|---|
| 1998 | Manatawny | 21.43 | 50.00 | 14.29 | 0.00 | 14.29 | 0.00 | 0.00 | 0.00 |
| | Perkiomen | 81.25 | 12.50 | 6.25 | 0.00 | 0.00 | 0.00 | 0.00 | 0.00 |
| | West Branch Perkiomen | 33.33 | 14.81 | 44.44 | 0.00 | 7.41 | 0.00 | 0.00 | 0.00 |
| | East Branch Perkiomen | 0.00 | 50.00 | 0.00 | 50.00 | 0.00 | 0.00 | 0.00 | 0.00 |
| | Skippack | 60.00 | 0.00 | 40.00 | 0.00 | 0.00 | 0.00 | 0.00 | 0.00 |
| | Wissahickon | 53.85 | 15.38 | 15.38 | 7.69 | 7.69 | 0.00 | 0.00 | 0.00 |
| 2010 | Manatawny | 100.00 | 0.00 | 0.00 | 0.00 | 0.00 | 0.00 | 0.00 | 0.00 |
| | Perkiomen | 57.14 | 4.76 | 38.10 | 0.00 | 0.00 | 0.00 | 0.00 | 0.00 |
| | West Branch Perkiomen | 35.71 | 14.29 | 42.86 | 7.14 | 0.00 | 0.00 | 0.00 | 0.00 |
| | East Branch Perkiomen | 83.33 | 0.00 | 16.67 | 0.00 | 0.00 | 0.00 | 0.00 | 0.00 |
| | Skippack | 88.57 | 5.71 | 5.71 | 0.00 | 0.00 | 0.00 | 0.00 | 0.00 |
| | Wissahickon | 38.71 | 3.23 | 58.06 | 0.00 | 0.00 | 0.00 | 0.00 | 0.00 |
| 2019 | Manatawny | 42.50 | 32.50 | 5.00 | 0.00 | 15.00 | 0.00 | 2.50 | 2.50 |
| | Perkiomen | 45.45 | 45.45 | 4.55 | 4.55 | 0.00 | 0.00 | 0.00 | 0.00 |
| | West Branch Perkiomen | 45.71 | 34.29 | 11.43 | 5.71 | 2.86 | 0.00 | 0.00 | 0.00 |
| | East Branch Perkiomen | 37.50 | 12.50 | 25.00 | 0.00 | 12.50 | 0.00 | 12.50 | 0.00 |
| | Skippack | 26.67 | 46.67 | 6.67 | 20.00 | 0.00 | 0.00 | 0.00 | 0.00 |
| | Wissahickon | 44.44 | 22.22 | 33.33 | 0.00 | 0.00 | 0.00 | 0.00 | 0.00 |
| | Laboratory Controls | 58.82 | 23.53 | 0.00 | 0.00 | 0.00 | 17.65 | 0.00 | 0.00 |

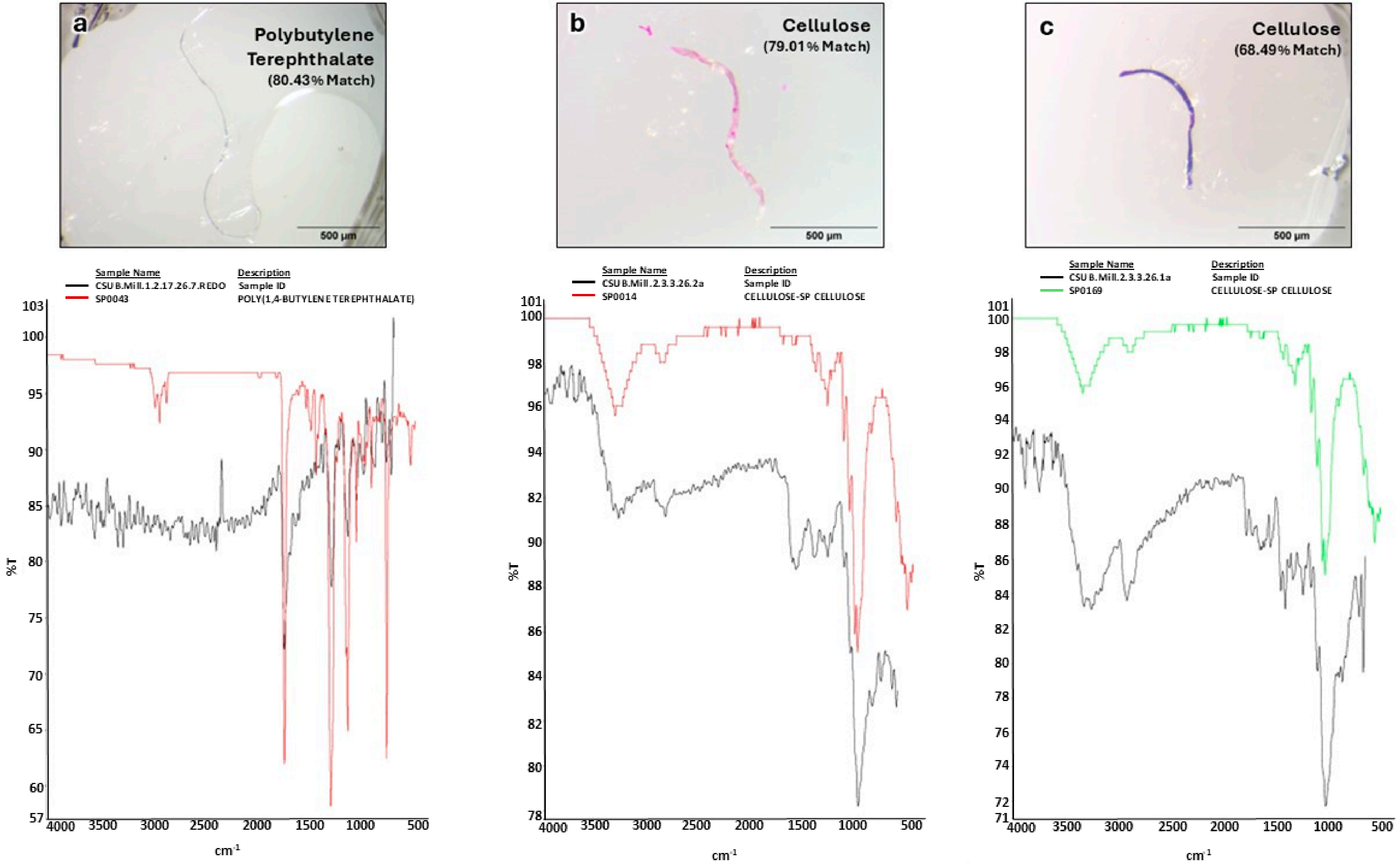


**Figure S1:** Examples of anthropogenic particles after Rose Bengal dye staining to differentially stain (a) microplastic and (b, c) natural-based anthropogenic particles with associated µ-FTIR spectra. Percent matches reported indicate sample material spectra (black lines) in comparison to reference library spectra (red & green line) matches. Note that the blue cellulose fiber (c) has a light purple tinted color due to the natural-based anthropogenic particle stained with the Rose Bengal over the fiver's blue pigment.

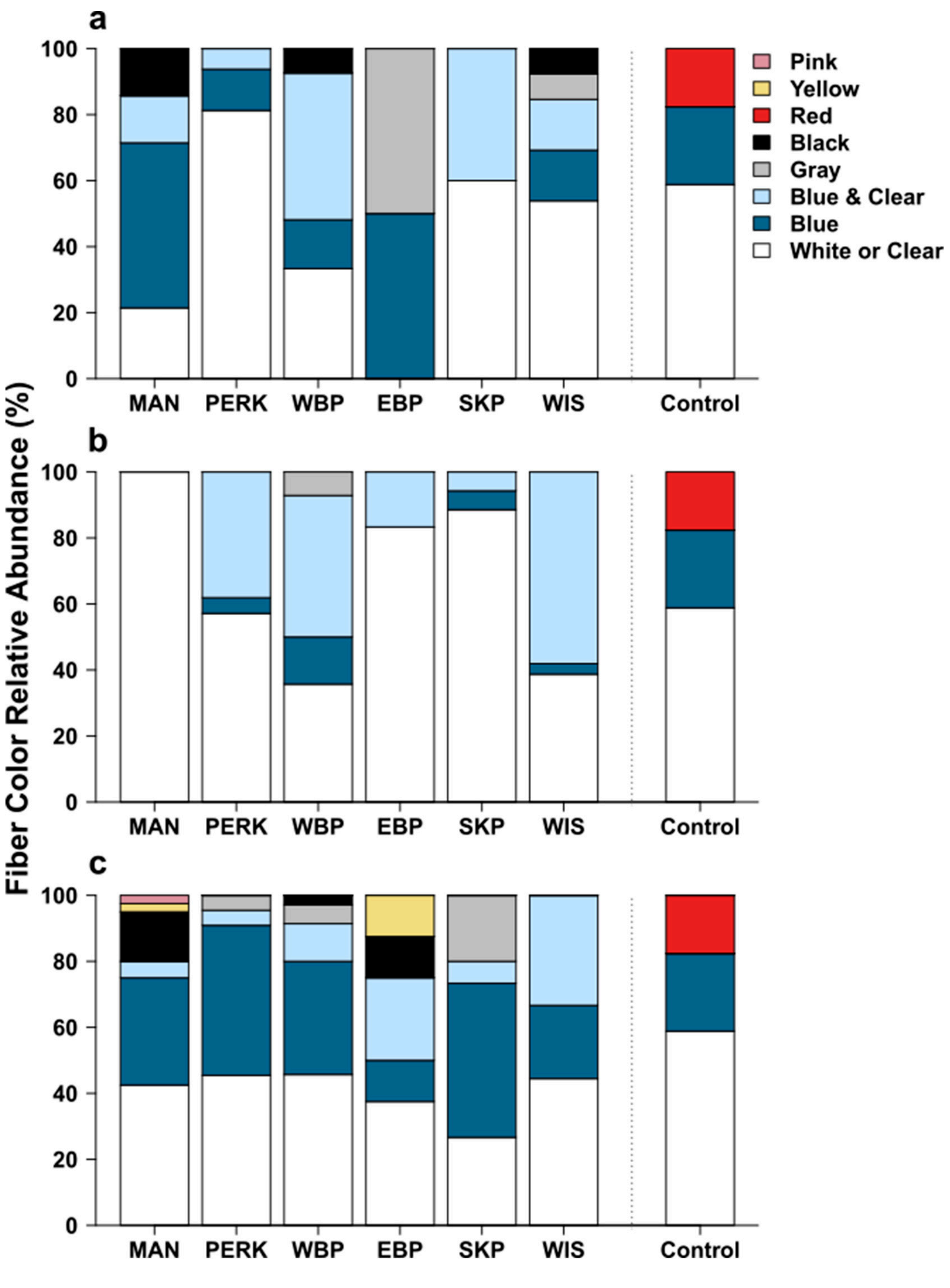


**Figure S2:** Relative abundance (%) of microplastic fiber color in environmental samples during (a) 1998, (b) 2010, and (c) 2019. Control data represents relative abundance of microplastics fibers from laboratory controls.

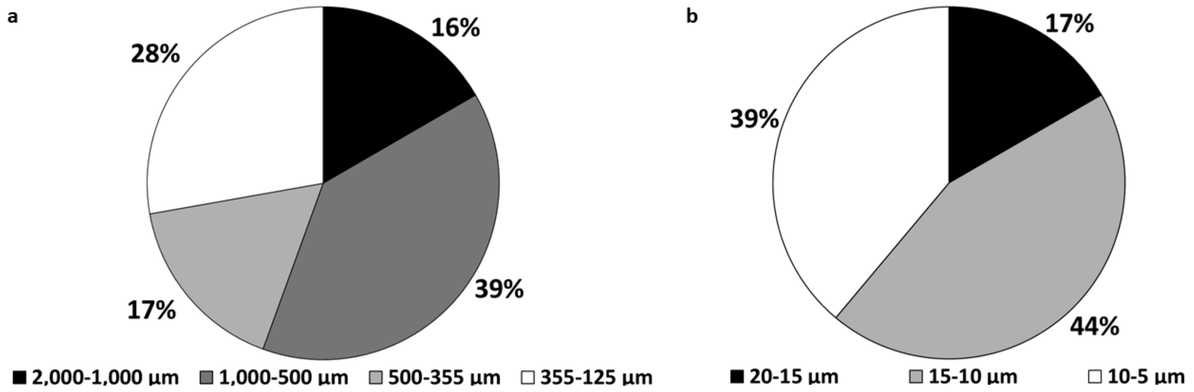


**Figure S3:** Microplastic fiber (a) length (µm) and (b) width (µm) distributions across a subset of particles picked from samples collected in 1998 and 2019 (*n* = 18).
